# Chromatographic and Spectral Analysis of Two Main Extractable Compounds Present in Aqueous Extracts of Laminated Aluminum Foil Used for Protecting LDPE-Filled Drug Vials

**DOI:** 10.1155/2009/693210

**Published:** 2009-09-23

**Authors:** Samuel O. Akapo, Sajid Syed, Anicia Mamangun, Wayne Skinner

**Affiliations:** ^1^Department of Analytical Development, Dey L.P., 2751 Napa Valley Corporate Drive, Napa, CA 94558-6268, USA; ^2^Department of Analytical Services, Teva Parenteral Medicines, Inc., 19 Hughes, Irvine, CA 92618-1902, USA

## Abstract

Laminated aluminum foils are increasingly being used to protect drug products packaged in semipermeable containers (e.g., low-density polyethylene (LDPE)) from degradation and/or evaporation. The direct contact of such materials with primary packaging containers may potentially lead to adulteration of the drug product by extractable or leachable compounds present in the closure system. In this paper, we described a simple and reliable HPLC method for analysis of an aqueous extract of laminated aluminum foil overwrap used for packaging LDPE vials filled with aqueous pharmaceutical formulations. By means of combined HPLC-UV, GC/MS, LC/MS/MS, and NMR spectroscopy, the two major compounds detected in the aqueous extracts of the representative commercial overwraps were identified as cyclic oligomers with molecular weights of 452 and 472 and are possibly formed from poly-condensation of the adhesive components, namely, isophthalic acid, adipic acid, and diethylene glycol. Lower molecular weight compounds that might be associated with the “building blocks” of these compounds were not detected in the aqueous extracts.

## 1. Introduction

The potential for adulteration of finished drug products by extractable and leachable compounds from the container or closure systems continues to receive greater attention and scrutiny by regulatory authorities. Consequently, numerous types of guidance including reviews have been published to assist drug manufacturers in providing adequate information regarding the identity, quantity, and control of such compounds to ensure the quality and/or safety of the drug product [1–4]. The focus of these documents is the requirement by the pharmaceutical industry to investigate both analytically and toxicologically compounds that may potentially leach from the packaging materials into the drug products [4, 5].

Several studies have been reported that characterize or identify extractables and leachables from plastic/rubber materials commonly used in packaging and drug delivery devices using various analytical techniques including chromatography, mass spectrometry, and organic synthesis [6–12]. Using both the GC/IR and GC/MS after isolation by Soxhlet extraction, Kim-Kang and Gilbert [13] identified seven unknown compounds that could migrate from plastic laminates into a unit dose injection device. However, little is known about extractables and leachables from preprinted laminated aluminum foil overwrap that is used for protecting drug products (e.g., inhalation solutions and suspensions) packaged in semipermeable containers (e.g., low-density polyethylene (LDPE)) from degradation and/or evaporation. 

 The components of the protective foil, which include inks, solvents, and unreacted monomers and oligomers derived from the adhesive material, have the potential to permeate through LDPE vials and contaminate the drug product formulations. While the identities of the inks and the associated volatile solvents are often known [14], the identities of compounds that may leach from other components of the packaging material and which may vary in structure depending on the nature of the finished drug product and the condition of use are not even known to the manufacturer. The goal of this study was to structurally identify the compounds obtained from an aqueous extract of preprinted foil laminate overwrap using chromatography, mass spectrometry and nuclear magnetic resonance (NMR) spectroscopic techniques.

## 2. Experimental

### 2.1. Materials and Reagents

Analytical grade phthalic acid, acetic acid, and hydrochloric acid were obtained from Mallinckrodt (Phillipsburg, NJ, USA) and used as received. HPLC grade acetonitrile and methanol were from EMD Chemicals (Gibbstown, NJ, USA). Adipic acid, 0.2 M trimethylphenylammonium hydroxide (TMAH) in methanol, and spectroscopy grade trifluoroacetic acid (TFA) were purchased from Sigma-Aldrich (St. Louis, MO, USA). The foil laminate overwrap was obtained from a commercial source, and for proprietary reasons, detailed information pertaining to this material and the drug product evaluated during the course of this study will not be disclosed. BondElut C_18_ solid-phase extraction cartridges (5 gm, 20 cc) were sourced from Varian (Lake Forest, CA, USA).

### 2.2. Sample Preparation

Extractable compounds from the laminated aluminum foil overwrap were extracted into 20 mL of purified water placed in a foil pouch (2 in. × 4 in.) previously rinsed twice with 20 mL of purified water. The pouch was sealed and incubated at 70°C for 24 hours in an oven. The pouched extract was then allowed to cool to room temperature and subsequently analyzed for any extractable compound from the foil.

### 2.3. HPLC

HPLC separation was performed using an Agilent 1100 series liquid chromatographic system (Wilmington, DE, USA) consisting of a quaternary gradient pump, heated column compartment, autosampler, photodiode array, and variable wavelength UV detectors. Data were collected and processed using a Perkin-Elmer Turbochrom Client/Server Data System, Version 6.1.2 Shelton, CT, USA. The separation employed an Agilent Zorbax, RX-C_18_ column (4.6 mm i.d. × 15 cm, 5 *μ*m particle size) (Wilmington, DE, USA) and the mobile phase consists of acetonitrile and 0.1% TFA in the ratio 38 : 62 v/v, filtered through a nylon membrane and degassed under vacuum before use. The column compartment was maintained at 25°C. Using a 50 *μ*L injection volume, the analytes were monitored with UV detection at 210 nm for a total runtime of 20 minutes at a flow rate of 1.0 mL/min.

**Figure 1:**
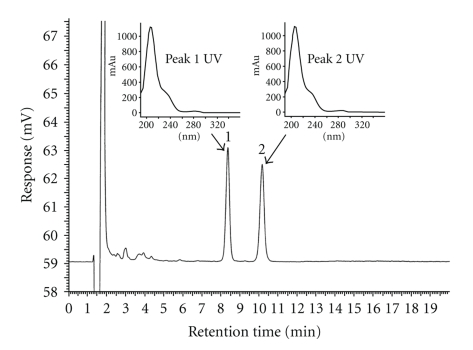
Chromatographic and UV profiles of extractable peaks **1** and **2** from aqueous laminated aluminum foil pouch extract.

 A typical chromatogram of the laminated foil extract is presented in Figure 1 showing two major extractable compounds, identified as peaks **1** and **2**, with the corresponding UV spectra (insert). The blank chromatogram (not shown) showed no peaks in the HPLC beside the solvent front. The retention times for triplicate analysis of the foil extract were 8.4 (0.4% R.S.D) and 10.2 (0.3% R.S.D) minutes, respectively, for peaks **1** and **2**, and the resolution (*R_s_*) between the two peaks was 4.6. The limits of detection and quantitation were determined to be 0.02 ppm and 0.06 ppm (1.4% RSD, *n* = 3), respectively, with the corresponding signal-to-noise ratios of approximately 3.8 and 9.9 [15]. Under the described HPLC conditions, several fractions of the extractable peaks **1** and **2** were collected and prepared for structural elucidation.

### 2.4. GC-MS

Separate HPLC fractions of the extractable peaks were dried using a Büchi Rotavapor R-124 rotary evaporator (Brinkmann Instruments, Inc., Westbury, NY), redissolved in 0.5 mL of 0.5 M methanolic HCl, and the resulting solution was heated for 1 hour at 70°C. The hydrolysates were then dried and dissolved in 40 *μ*L of 0.2 M TMAH in methanol to produce the methyl derivatives, which were subsequently analyzed using Agilent 6890/5973 gas chromatograph-mass spectrometry detector, GC-MSD (Wilmington, DE) equipped with a Gerstel MultiPurposeSampler MPS (Baltimore, MD). The gas chromatograph was fitted with Agilent DB-5MS capillary column (0.25 *μ*m × 30 m, 0.25 *μ* film) and operated with temperature programming from 50°C (held for 1 min) to 300°C at 10°C/minutes, and held at 300°C for 6 min using helium as the carrier gas at a constant flow rate of 1 mL/min. The GC injector port was set at 280°C in a splitless mode and the MSD was maintained at 280°C. All the GC/MS data were acquired with MSD ChemStation Version D.01.02.16 (Agilent Technologies, Wilmington, DE) in the *m/z* range of 30–500 at a rate of 1 scan/sec under electron ionization (EI) mode.

### 2.5. LC-MS

A ThermoSeparations HPLC pump (Model P400) and UV detector (Model 600LP) coupled to a Finnigan LCQ Duo ion-trap mass spectrometer with electrospray source (ThermoQuest, San Jose, CA, USA) were used to obtain full-scan MS and MS/MS data of the foil laminate extractables. The column and conditions of HPLC analysis were as described in HPLC section except the mobile phase, which contains 40 : 60 v/v acetonitrile: 0.1% acetic acid. Foil laminate extract was analyzed by LC/MS in full-scan positive-ion mode using a 50 to 1500 *m/z* scan range. Following the assignment of MS ions for the identified peaks, the extract was reanalyzed using full-scan MS/MS experiments to obtain product ion spectra.

### 2.6. NMR

Further confirmation of the structure of each extractable compound was performed on a JOEL ECX-400 NMR spectrometer operating at 400 MHz at Acorn NMR Incorporated (Livermore, CA, USA) after isolation using solid-phase extraction (SPE) followed by analytical HPLC purification. For SPE, a BondElut C_18_ cartridge was washed with 0.1% TFA in methanol and preconditioned with 0.1% TFA in water before loading about 50 mL of the foil extract at approximately 3 mL/min. The retained compounds were eluted with 10 mL of 0.1% TFA in methanol, and the solvent was evaporated to dryness using the Büchi Rotavapor R-124 rotary evaporator. The residue was then redissolved in 0.2 mL methanol and rinsed with 0.8 mL of 0.1% TFA in purified water prior to HPLC purification of the isolates, which were subsequently redissolved in deuterated methanol (CD_3_OD) containing tetramethylsilane (TMS) for NMR analysis. ^1^H and COSY spectra were acquired at ambient temperature (25°C), and the resulting FIDs were transferred to a computer and processed using NutsPro NMR software (Acorn NMR Inc., Livermore, CA, USA). ^1^H chemical shifts were referenced to internal TMS.

## 3. Results and Discussion

### 3.1. GC-MS Profiles of Extracted Compounds

**Figure 2:**
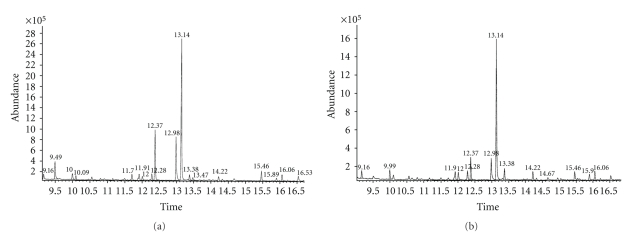
Total ion current GC/MS chromatograms of methylated derivatives of hydrolyzed (a) extractable **1** and (b) extractable **2**.

**Figure 3:**
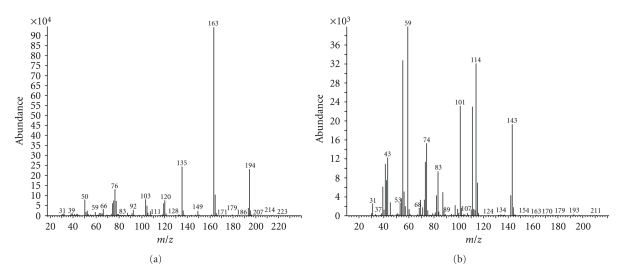
GC/MS spectral for peaks at (a) 13.14 min, and (b) 9.49 min in Figure 2(a).

The MS total ion chromatograms (TICs) for methylated fractions of hydrolyzed peaks **1** and **2** as illustrated in Figure 2 showed the presence of 3-4 major peaks in addition to several other minor peaks. The signal at *m/z* 194, 163, and 135 in Figure 3(a) for the main peak at about 13.14 minutes in Figure 2 matched the NIST mass spectral library for 1,3-dimethyl phthalate indicative of a strong preference for isophthalic acid (1,3-benzenedicarboxylic acid) in both the extractable compounds. Other peaks at about 12.37 and 12.98 minutes are identified as phthalic (1,2-benzenedicarboxylic acid) and terephthalic (1,4-benzenedicarboxylic acid) acids and are probably present in trace amounts in the starting materials. Although a fair majority of the peaks observed are very similar for the two compounds, one noticeable difference is the peak at about 9.49 minutes in Figure 2(a) for extractable **1**, which was absent in Figure 2(b) for extractable **2**. The signal at *m/z* 143, 114, 101, and 59 in Figure 3(b), which are characteristic of adipic acid, gave a good library match for 1,6-dimethyl hexanoate indicating the presence of adipic acid in extractable **1** alone.

### 3.2. LC-MS Analysis of Extracted Compounds

**Figure 4:**
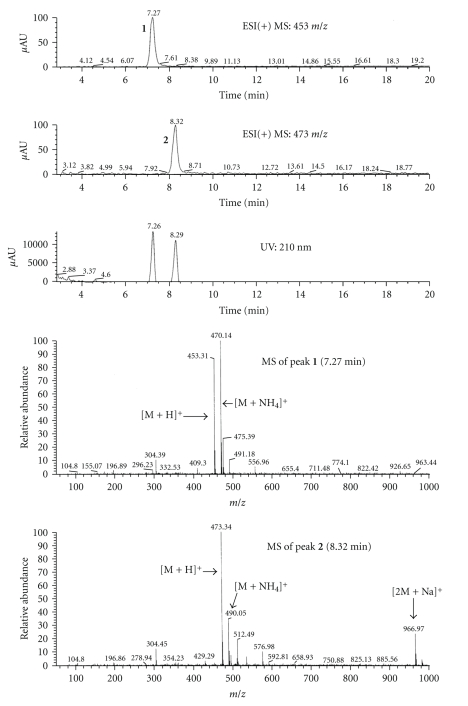
Full scan LC/MS spectrum for extractable peaks **1** and **2**.

The full scan MS spectra of extractables **1** and **2** are shown in Figure 4. The peak at 7.3 minutes produced a base peak at *m/z* 453 corresponding to the [M+H]^+^ ion and an ion due to the ammonia adduct [M+NH_4_]^+^ at *m/z* 470. The peak at 8.3 minutes produced a base peak at *m/z* 473 corresponding to the [M+H]^+^ ion, an ion at *m/z* 490 due to the ammonia adduct [M+NH_4_]^+^, and a sodium adduct of a dimer ion [2M+Na]^+^ at *m/z* 967. These full scan spectra enabled molecular weight assignments of 452 and 472 for extractables **1 **and **2**, respectively. 

**Figure 5:**
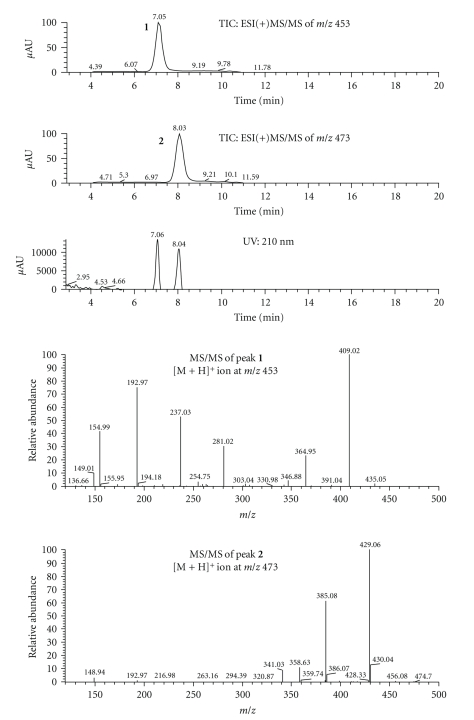
Full scan MS/MS spectrum for extractable peaks **1** and **2**.

Figure 5 shows the full scan MS/MS spectra of extractables **1** and **2**. The *m/z* 409 ion originating from pseudomolecular ion, [M+H]^+^, of *m/z* 453 for extractable **1** represents a loss of C_2_H_4_O yielding the *m/z* 409 ion. Additional elimination of CO_2_ (44 u) and C_5_H_8_O (84 u) yielded ions with *m/z* 365 and 281, respectively. Further cleavage of C–O bonds produced ions with *m/z* 237 and *m/z* 193, and the latter produced *m/z* 149 ion after the loss of C_2_H_4_O and intramolecular cyclization. On the other hand, after loss of C_2_H_4_O and CO_2_ from molecular ion, [M+H]^+^, of *m/z* 473 for extractable **2**, fragment ions *m/z* 429 and *m/z* 385 were formed, respectively. Subsequently, loss of the C_2_H_2_ molecule (26 u) from fragment ion *m/z* 385 yielded the *m/z* 359 ion. A comparison of the molecular weights for the two compounds gave a difference of 20 u indicating a possible replacement of an isophthalic acid molecule with adipic acid in extractable **1**.

**Figure 6:**
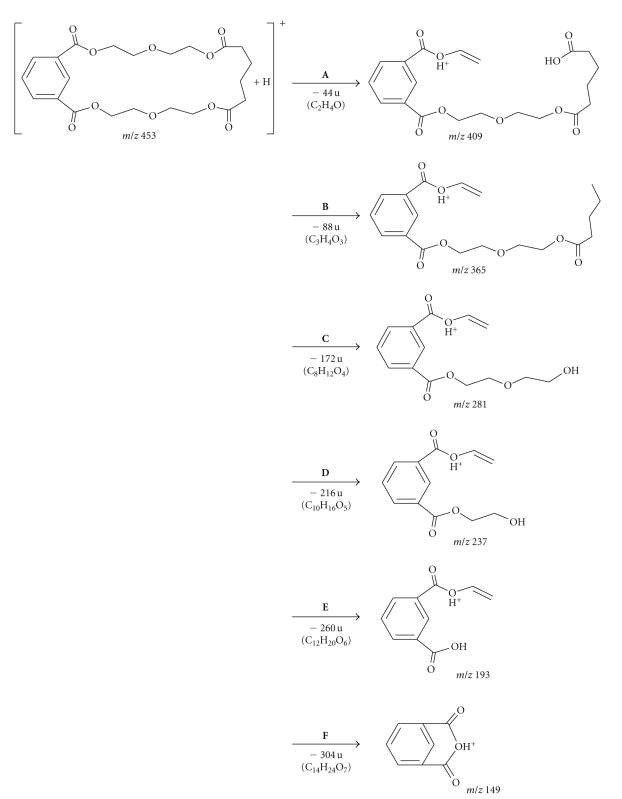
Proposed structure and mass spectrometric fragmentation pathway of extractable peak **1**.

**Figure 7:**
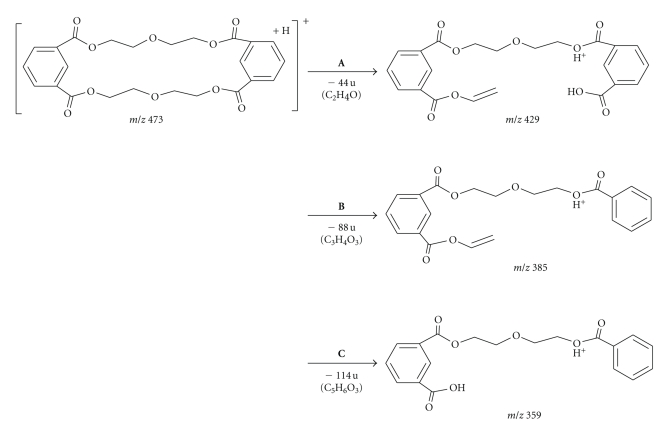
Proposed structure and mass spectrometric fragmentation pathway of extractable peak **2**.

 The LC/MS results were in agreement with the GC/MS data, in which both compounds contained the isophthalic acid moiety whereas the adipic acid moiety was only detected in extractable **1**. Thus, extractable **1** appears to be a reaction product of a molecule each of isophthalic and adipic acids with two molecules of diethylene glycol, while extractable **2** is formed by poly-condensation of two molecules of isophthalic acid with two diethylene glycol molecules. The proposed structures and the fragmentation patterns for the two compounds are shown in Figures 6 and 7. Additionally, the MS/MS experiments on the ammonia adducts of extractable peaks **1** and **2** produced only the loss of NH_3_ to the corresponding pseudomolecular ions, confirming the adduct assignments (data not shown).

### 3.3. NMR Spectroscopic Analysis for Accurate Structure Determination of Peaks **1** and **2**

**Figure 8:**
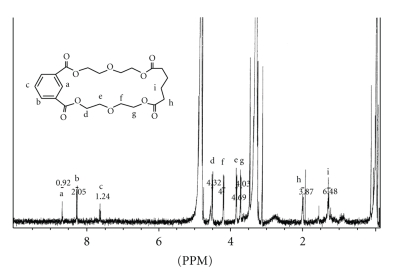
The ^1^H NMR (400 MHz) spectrum for extractable peak **1** in CD_3_OD.

**Figure 9:**
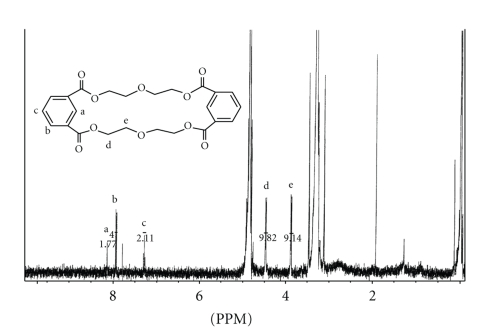
The ^1^H NMR (400 MHz) spectrum for extractable peak **2** in CD_3_OD.

The isolated and purified fractions of the two extractable compounds were analyzed by ^1^H NMR spectroscopy to provide further verification of the proposed structures. The ^1^H NMR spectra of the extractable compounds **1 **and **2 **are shown in Figures 8 and 9, respectively. In addition to several peaks, each spectrum showed the expected solvent peaks at *δ* 4.9 and 3.3 ppm for HDO and CD_2_HOD, respectively. In Figure 8, the three aromatic protons (a, b, and c) could easily be found at *δ* 8.68, 8.28, and 7.65 ppm as triplet, doublet of doublets, and triplet, respectively. The coupling patterns and the magnitude of the coupling constants are characteristic of a *meta*substitution. The methylene protons d and g, next to the ester groups, appeared at *δ* 4.52 and 3.73 ppm as complex multiplets, respectively, the shifts of which are consistent with the presence of the isophthalate unit. Protons d were assigned *δ* 4.52 ppm, the most downfield of the pair, as they are esters of the aromatic isophthalic acid group. The resonances of the methylene protons e and f appeared at *δ* 3.84 and 4.20 ppm, respectively, as complex multiplets. The splitting patterns of protons d, e, f, and g are typical of X-CH_2_CH_2_-Y spin systems. The chemical shift for protons h was observed at *δ* 2.00 ppm, typical of a methylene *alpha* to a carbonyl, while protons i was observed at *δ* ~ 1.3 ppm, typical of a methylene *beta* to a carbonyl. The COSY spectrum (not shown) demonstrated protons d to be coupled to e, f to be coupled to g, and finally h to be coupled to i.

 The ^1^H NMR spectrum of extractable **2** could also be clearly assigned as shown in Figure 9. The resonances of the three aromatic protons (a, b, and c) are similar but appear slightly more upfield than those in extractable **1**. The methylene protons d and e appeared at *δ* 4.48 and 3.89 ppm, respectively, as complex multiplets, as would be expected for ethylene glycols. These shifts and splitting patterns are similar to the analogous protons d and e of extractable **1**. Redundant ^1^H positions in both structures were not labeled due to symmetry. The absence of vinylic and/or carboxylic protons and the fact that the molecules clearly exhibited elements of symmetry, indicate that both extractables are nonlinear contrary to the structures reported by Tiller et al. [12] for two leachable compounds obtained from a custom adhesive used during development of a medical device. Therefore, we propose that extractable **1** is a cyclic oligomer of isophthalic, adipic acid, and diethylene glycol, and extractable **2** is a cyclic oligomer of isophthalic acid and diethylene glycol.

## 4. Conclusion

The two compounds detected in the aqueous extract from laminated aluminum foil overwrap were structurally identified as cyclic oligomers of (i) isophthalic, adipic acid and diethylene glycol, and (ii) isophthalic acid and diethylene glycol, with molecular weights of 452 and 472, respectively, using combined GC/MS, LC/MS/MS and NMR spectroscopy. Presumably due to lack of chromophoric functional groups, lower molecular weight and thus more water soluble cyclic compounds that might be associated with the building blocks of the two compounds were not detected in the aqueous extracts of the aluminum foil examined. While a discussion concerning the potential toxicity of these compounds is beyond the scope of this paper, laminated aluminum foils have been certified to be used as packaging materials for LDPE-filled drug vials at our facility through extensive stability studies for several clinical, registration, and commercial batches of aqueous-based medications. Data from these studies have shown that these compounds are either completely absent or present in the drug products at or below the detection limit of the test method (DL <0.02 ppm), which is also lower than the FDA/ICH threshold of ≤1.0% for impurities in new drug products [16].
